# Hepatic von Meyenburg complex: a trigger of severe portal hypertension

**DOI:** 10.1111/j.1478-3231.2008.01903.x

**Published:** 2009-04

**Authors:** Shuhei Yoshida, Kazutaka Kurokohchi, Takuya Ueno, Morihiko Yoshino, Masahiko Shimada, Tsutomu Masaki

**Affiliations:** 1 Department of Gastroenterology, Internal Medicine, TMG Asakadai Central General HospitalSaitama, Japan; 2 Division of Gastroenterology and Hepatology, Internal Medicine, Kagawa University School of MedicineKagawa, Japan; 3 Critical Care Medicine, Hachioji Medical Center, Tokyo Medical UniversityTokyo, Japan

To the Editor:

Multiple bile duct hamartoma [von Meyenburg complex (VMC)] is a benign liver malformation that includes biliary cystic lesions with congenital hepatic fibrosis causing ductal plate malformations (1–7). It is generally asymptomatic and tends to be identified either at autopsy or during histological examinations (1). We report a very rare case of severe portal hypertension caused by VMC diagnosed by magnetic resonance cholangiopancreatography (MRCP) and histopathology.

## Case report

**Fig. 1:**
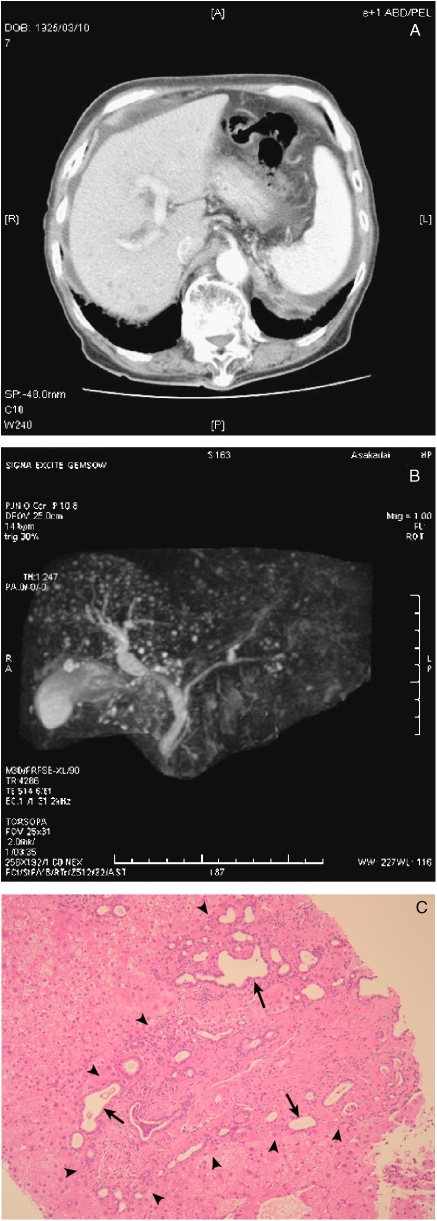
(A) Axial computed tomography 42 × 42 mm (300 × 300 DPI). (B) T2-weighted magnetic resonance cholangiogram shows numerous high-signal-intensity liver lesions and no communication between these and the normal-sized intra- and extrahepatic biliary system. 42 × 42 mm (300 × 300 DPI). (C) Photomicrograph (original magnification, × 40; H&E stain) shows a portal region (arrowheads) containing several cystic spaces, which are interspersed with fibrous stroma and lined by a layer of biliary epithelium (arrows). 84 × 63 mm (300 × 300 DPI).

An 88-year-old female was hospitalized with hypoxic orthopnoea, abdominal fullness and leg oedema. Arterial blood gas analysis revealed 88% oxygen saturation. A chest X-ray revealed a widespread right pleural effusion. Past history was unremarkable. Laboratory tests showed normal liver chemistry except for mild elevations of alkaline phosphatase and γ-glutamyl transpeptidase. Antinuclear antibody and antimitochondrial-2 antibody were negative. Serology for hepatitis B virus, hepatitis C virus and HIV were negative. Tumour markers showed: α-foetoprotein, 2.4 ng/ml (0–20 ng/ml); carbohydrate antigen 19-9, 56.6 U/ml (0–37 U/ml); and carcinoembryonic antigen, 9.0 ng/ml (0–5.0 ng/ml). Endoscopy revealed large oesophageal varices in the mid-oesophagus. Abdominal computed tomography revealed multiple cystic images in the whole liver and both kidneys, some ascites and mild splenomegaly (Fig. 1A). The intrahepatic and extrahepatic bile ducts were not dilated. No evidence of cirrhosis, heart failure or renal dysfunction was observed. Especially, brain natriuretic peptide, human atrial natriuretic peptide and cardiac ejection fraction were normal. Furthermore, no malignant lesion or vessel thrombus was seen. MRCP T2-weighted sequences showed multiple small hyperintense lesions <1 cm diameter and normal intrahepatic and extrahepatic bile ducts (Fig. 1B). Inferior vena cavography and hepatic venography showed no angiostenosis or thrombus in the major blood vessels. Hepatic venous pressure gradient was 14 mmHg. Therefore, VMC or multiple liver cysts were strongly suspected. A liver biopsy revealed diffuse bile duct hamartomas arising in the portal region. Haematoxylin–eosin staining showed bile duct microhamartomas consisting of circumscribed fibrous areas containing many irregularly dilated bile duct structures and only a few narrowed vessels in the portal region (Fig. 1C). A narrowing of the portal vein might have occurred. Therefore, a diagnosis of VMC with portal hypertension was confirmed. We believe that the displacement of the portal vein triggered portal hypertension. Furosemide therapy rapidly improved the patient's condition. One year later, the patient's condition has remained stable on diuretic treatment. However, a follow-up endoscopy showed mid-oesophageal varices unchanged from the previous.

## Discussion

This is the first report of a VMC patient with severe symptoms due to portal hypertension. Magnetic resonance imaging is generally useful for the diagnosis of VMC (2, 3). This condition is incidentally detected in from 0.5 to 5.6% of autopsies (1) and is generally asymptomatic. In contrast, our patient presented with severe portal hypertension. It was thought that portal hypertension was due to displacement of the portal vein by the presence of VMC. As a result, severe pleural effusion, ascites and oesophageal varices developed. Previous reports described VMC cases with epigastric pain (4, 5). However, those were not pure VMC cases.

In contrast, there are only a few published VMC reports supporting the current findings (5–7). Brancatelli *et al*. (7) described portal hypertension that occurred from fibropolycystic liver disease. Their observations are helpful for our clinical perspective. Despite furosemide treatment, oesophageal varices remained unchanged, indicating the presence of persisting portal hypertension in the setting of VMC.

In conclusion, VMC can trigger severe symptoms requiring urgent and continuous treatment. The present case therefore fell outside of the traditional diagnostic criteria and management guidelines for VMC. These findings suggest that the concept of VMC may require modification because it does not apply to all cases. In addition, the clinicopathological and radiological features of VMC, as well as the pathogenesis need further study and clarification.
